# Exome sequencing identifies novel compound heterozygous IFNA4 and IFNA10 mutations as a cause of impaired function in Crohn’s disease patients

**DOI:** 10.1038/srep10514

**Published:** 2015-05-22

**Authors:** Chuan-Xing Xiao, Jing-Jing Xiao, Hong-Zhi Xu, Huan-Huan Wang, Xu Chen, Yuan-Sheng Liu, Ping Li, Ying Shi, Yong-Zhan Nie, Shao Li, Kai-Chun Wu, Zhan-Ju Liu, Jian-Lin Ren, Bayasi Guleng

**Affiliations:** 1 Department of Gastroenterology, Zhongshan Hospital affiliated to Xiamen University, 201 Hubin South Road, Xiamen, Fujian Province, 361004, China; 2 Faculty of Clinical Medicine, Medical College of Xiamen University, 168 University Road, Xiamen, Fujian Province, 361005, China; 3 State Key Laboratory of Cellular Stress Biology, Xiamen University, 168 University Road, Xiamen, Fujian Province, 361005, China; 4 BGI-Shenzhen, Shenzhen, Guangdong, 518083, China; 5 Department of Gastroenterology, Shanghai Tenth People’s Hospital, Tongji University, Shanghai 200072, China; 6 State Key Laboratory of Cancer Biology and Xijing Hospital of Digestive Diseases, Fourth Military Medical University, West Changle Road 15, Xi’an, 710032, China; 7 MOE Key Laboratory of Bioinformatics and Bioinformatics, Tsinghua University, Beijing, 100084, China

## Abstract

Previous studies have highlighted the role of genetic predispositions in disease, and several genes had been identified as important in Crohn’s disease (CD). However, many of these genes are likely rare and not associated with susceptibility in Chinese CD patients. We found 294 shared identical variants in the CD patients of which 26 were validated by Sanger sequencing. Two heterozygous IFN variants (IFNA10 c.60 T > A; IFNA4 c.60 A > T) were identified as significantly associated with CD susceptibility. The single-nucleotide changes alter a cysteine situated before the signal peptide cleavage site to a stop code (TGA) in IFNA10 result in the serum levels of IFNA10 were significantly decreased in the CD patients compared to the controls. Furthermore, the IFNA10 and IFNA4 mutants resulted in an impairment of the suppression of HCV RNA replication in HuH7 cells, and the administration of the recombinant IFN subtypes restored DSS-induced colonic inflammation through the upregulation of CD4^+^ Treg cells. We identified heterozygous IFNA10 and IFNA4 variants as a cause of impaired function and CD susceptibility genes in Chinese patients from multiple center based study. These findings might provide clues in the understanding of the genetic heterogeneity of CD and lead to better screening and improved treatment.

Pathogenesis of Crohn’s disease (CD) is proposed to result from interactions between a genetic predisposition, environmental triggers, and mucosal immunity^1^,^2^,^3^. In recent years, genome-wide association studies (GWASs) have been applied with great success in identifying numerous candidate genes involved in CD, including TNFSF15^4^, IL23R^5^, ATG16L1^6^,^7^, the gene desert region on chromosome 5p13.1^8^, and IRGM^9^,^10^. Although a meta-analysis of 6 GWASs reported 163 CD susceptibility loci in the European population, those loci were estimated to explain only 23.2% of the observed heritability^11^, indicating that a large number of CD susceptibility loci have not yet been identified^12^. Studies have shown apparent ethnic differences between European and East-Asian populations with regard to CD susceptibility loci, such as NOD2, which has been associated with CD in the USA and Western Europe but was not reported to be correlated with CD in such Asian populations as the Japanese, Chinese, and Korean populations^13^,^14^,^15^,^16^,^17^,^18^,^19^,^20^. Additionally, NOD2 was not correlated with some European populations, for example, Tunisians^21^.

Such genes with repeat evidence for a strong association suggest that pathways involving the disruption of the innate and adaptive immune systems, compromised epithelial barrier function, and impaired autophagy play a significant role in the relevant disease^1^. However, despite the identification of more than 100 unique genes in inflammatory bowel disease (IBD) susceptibility, these common variants combined account for less than a quarter of the genetic risk^11^,^22^. Indeed, rare variants form the group of infrequent mutations that occur in <5% of the population; a large proportion of variants in this class occur at a much lower frequency (<0.1%), and many thousands are likely to be specific to ethnic groups, communities, families, or even individuals^23^. Furthermore, evidence of causality for specific variants is largely absent.

Currently, whole-exome capture based on high-throughput sequencing technology provides a powerful and affordable means to identify causative variants^24^. Exome sequencing has already proven to be successful in identifying causal mutations in an ever-growing list of both recessive and dominant rare Mendelian disorders, whereby the sequencing of a small number of unrelated cases has been used to identify disease-causing variants^24^. One such case involved the effective clinical application of whole-exome sequencing in a child with intractable IBD, an approach that successfully identified a causal mutation in the XIAP gene, with hematopoietic progenitor cell transplant treatment, as recommended for XIAP deficiency, resolving the IBD^25^. Another study reported the exome sequencing of pediatric IBD patients, identifying rare and novel variants in known IBD susceptibility genes^23^.

However, the variation associated with CD identified in Western populations might be rare and not associated with susceptibility to CD in Chinese patients of the Han population, suggesting the possible genetic heterogeneity of CD in different populations. Moreover, no specific susceptible genes have been reported to date in Chinese CD patients. In this study, we applied exome sequencing in four CD individuals, and verified the variants using Sanger sequencing in an expanded group of CD cases and healthy individuals. We identified heterozygous IFNA10 and IFNA4 variants as a cause of impaired function and CD susceptibility genes in the Chinese population from multiple center based study. These findings might provide clue for the understanding of the genetic heterogeneity of CD and lead to better screening and improved treatment.

## Result

### Exome capture and sequencing

We prepared an exome library of four individuals clinically diagnosed as refractory CD (Supplementary Table 1). Each captured library was hybridized to the Sure Select Biotinylated RNA Library (BAITS) for enrichment and then loaded onto the Hiseq2000 platform. We sequenced an average of 5.57 Gb with a 75.60 × mean depth for per affected individual as 90 bp paired-end reads. We achieved approximately 97.91% targeted base coverage, which is sufficient for variant calling (Supplementary Table 2).

### Variants in CD exome

We obtained 62091, 63581, 58329, and 59449 SNPs and 5192, 5112, 4983, and 5025 indels by using SOAPaligner. And SOAPsnp2 of the exon sequences from the four affected individuals (Supplementary Table 3). To identify the potential causative mutations among these variants, we focused on three types of variants that are more likely to be pathogenic than other variants: non-synonymous (NS) variants, splice acceptor and donor site mutations (SS), and short, frame-shift coding insertions or deletions (indels). Thus, we selected NS/SS/indel variants present in at least one of the four CD-affected individuals, and the single-nucleotide variations were filtered using the dbSNP (v129), 1000 Genomes Project(1000genomes release_20100804), HapMap databases((2010-08_phaseII + III)), and YH databases. As a result, we identified 1648, 1632, 1602, and 1623 variants in the four affected individuals (Supplementary Table 4).

Hypothesizing that the CD-affected individuals share the same causal variant, we compared the exome data from the four samples to identify shared variants and candidate genes, obtaining 294 genes with complete identical variants (Supplementary Table 4). Considering that most pathogenic variants either affect highly conserved sequences and/or are predicted to be deleterious, we used SIFT to predict the functional impact of the variants. As a result, 57 candidate genes were validated by Sanger sequencing (Supplementary Table 4).

### Validation of the candidate gene variants with Sanger sequencing

We performed Sanger sequencing to further validate 57 putative variants identified in the four affected individuals. After filtering out the genes for which the variants were not validated by Sanger sequencing or were not cloned by PCR, a total of 26 genes were confirmed and represented in all four affected individuals (Supplementary Table 6). Sanger sequencing for these 26 gene variants was then performed using an expanded group of 208 CD patients and 198 healthy individuals from three of medical center. Our results indicated that the heterozygous mutation frequency of IFNA10 rs10119910 (c.60 T > A) and IFNA4 rs113055208 (c.60 A > T) was significantly increased in CD patients compared to the healthy controls (Table 1, IFNA4, *P* = 0.005; IFNA10, *P* = 0.001). In addition, most of the CD cases were IFNA4/10 co-mutated, though no significant difference was observed between the IFNA single subtype and dual subtype variants (date was not shown). No significant differences were observed in the other 24 genes in this cohort (data not shown). Thus, we identified two heterozygous IFN variants as significantly associated with susceptibility to CD in the Chinese population from multiple center based analysis (Supplementary Table 7).

### Association of variants with Genotype-phenotype and medical treatment efficacy

When stratifying IBD patients according to the Montreal classification, we did not find a significant association between the frequencies of the two above alleles and clinical features, such as sex, age, disease localization or disease behavior (Supplementary Table 8 and Supplementary Table 9). However, we further explored the association of the genotypes with response to medical treatment in Chinese CD patients, and our results revealed significant differences in the IFNA4 and IFNA10 genotype frequencies between effective and ineffective responses to steroid treatment (Table 2 and Table 3, *P* < 0.05). Thus, we identified two heterozygous IFN variants in CD patients that are significantly associated with resistance to steroid treatment.

### Functional analysis of IFNA4 and IFNA10 variants ***in vitro***

**Figure 1:**
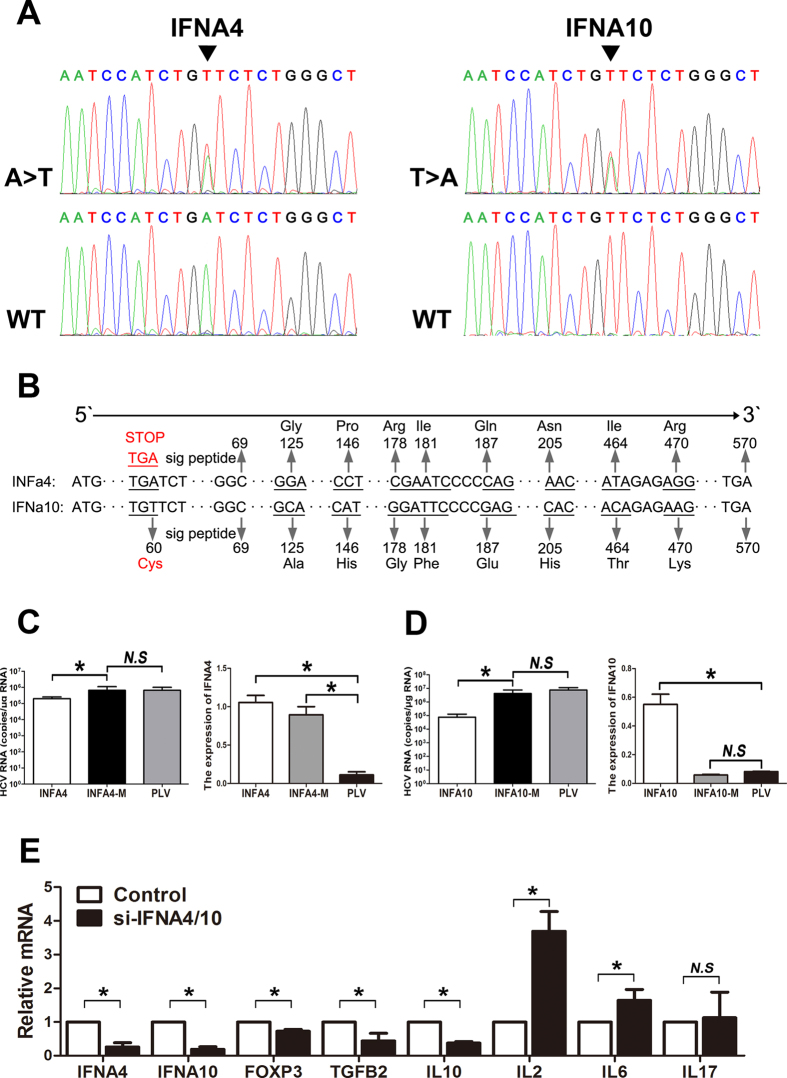
Sequencing and functional analysis of the IFNA4 and IFNA10 variants. (**A**) Confirmation of the IFNA4 and IFNA10 heterozygous mutations in CD patients compared to healthy subjects (wild-type alleles) using Sanger sequencing. (**B**) The mutation site and amino acids alteration of the IFNA4 and IFNA10 variants. (**C-D**) Huh7 cells were transiently transfected with plasmids expressing IFNA4, IFNA10, or mutants and then infected with HCV; the replicon copies were calculated using real-time PCR and PLV as a control; The supernatants of the cell culture were analyzed using ELISA. (**E**) Real-time PCR analyzed the mRNA expressions of IFNA4, IFNA10, FOXP3, TGFβ, IL10, IL2, IL6 and IL17 in NCM460 cells after transfection of the siRNA targeting IFNA4 and IFNA10. The scramble siRNA sequences were used as a negative control. The results indicate the mean ± SD of three independent experiments (*P < 0.05).

We further analyzed the characteristics of the IFNA4 and IFNA10 variants and found that the single-nucleotide changes of INFA10 (c.60 T > A) in exon 1 resulted in the alteration to a stop codon of a cysteine located before the signal peptide cleavage site. We hypothesized the A was wild-type allele for Chinese people, T was the mutant in IFNA4 and analyzed in opposite way. Then, observed the IFNA4 (c.60 A > T) variant induced the elongated IFNA4 (Fig. 1A,B). Both of variants may potentially impair IFN subtype function. Type 1 interferon (IFNA/B) is the first line of defense against viral infection, including HCV, and HCV replicons are highly sensitive to IFNA *in vitro*. To further determine the functional affects of the IFNA4 and IFNA10 variants, we transfected wild-type and mutant plasmids of IFNA4 and IFNA10 into HuH-7 cells infected with HCV. Our data demonstrated that the suppressive effects on HCV RNA replication were significantly attenuated with the transfection of the IFNA4 and IFNA10 mutants compared to the wild-type controls, indicating that the IFNA4 and IFNA10 variants impaired the suppression of HCV replication (Fig. 1C,D and Supplementary Figure 1). To investigate whether the IFNA4 and IFNA10 affect the Treg cell maturation, we knocked down the IFNA4 and IFNA10 in the colon cell of NCM460 via siRNA *in vitro*. We found that silencing the both of them induced the decrease of FOXP3, IL10, TGFβ and increase of IL2, IL6 expressions (Fig. 1E).

### IFNA4 and IFNA10 variant correlated with their serum level in CD patients

**Figure 2:**
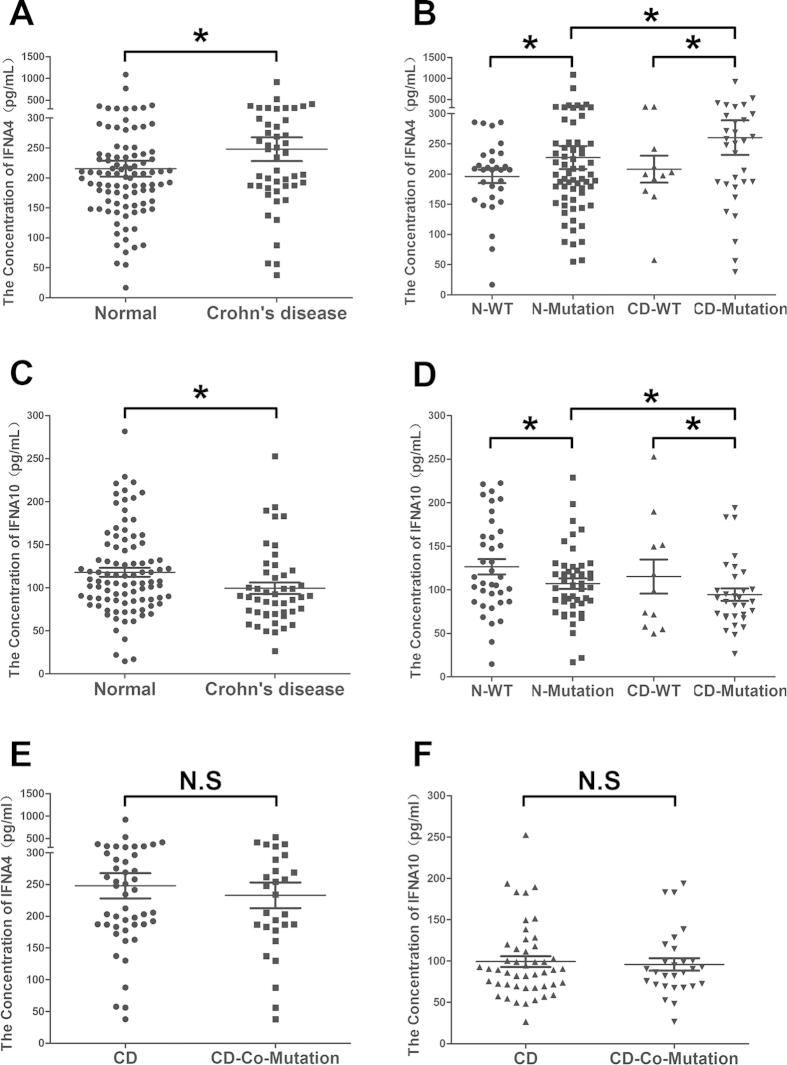
ELISA analysis of serum IFNA4 and IFNA10 levels. A Wilcoxon two-sample test was performed to evaluate the differences between 47 CD patients and 95 healthy controls. Comparison of the serum IFNA4 level between CD patients and healthy controls (**A**) and between wild-type and mutant alleles in CD patients and healthy controls (**B**) N indicates normal healthy control. Comparison of the serum IFNA10 level between CD patients and healthy controls (**C**) and between wild-type and mutant alleles in CD patients and healthy controls (**D**). (**E-F**) Comparison of serum IFNA4 and IFNA10 between IFN single-subtype and dual-subtype variants in CD patients, respectively (*P < 0.05).

To investigate whether the serum level of IFNA4 and IFNA10 can be correlated with the identified mutation, we measured the levels of the proteins in the sera of 47 patients with CD and 95 healthy individuals by ELISA. Our results indicated that the serum level of IFNA10 was significantly decreased in the CD patients compared to the healthy controls (Fig. 2C, 117.9 ± 5.17 vs. 99.4 ± 6.54, *P* < 0.05). The level of IFNA10 was also similarly decreased in individuals with the variant compared to the wild-type allele in both the CD and healthy control groups (Fig. 2D, *P* < 0.05) and was significantly decreased in the variant CD group compared to the variant control group. In contrast, the IFNA4 serum level was increased in the CD patients compared to the healthy controls (Fig. 2A, 215.3 ± 13.28 vs. 248 ± 19.86, *P* < 0.05), and the level of IFNA4 was also increased in the individuals with the variant compared to the wild-type allele (Fig. 2B, *P* < 0.05). In addition, no significant difference between the IFNA4 and IFNA10 levels were detected between the IFNA single-subtype and dual-subtype variant CD patients (Fig. 2E,F).

### IFNA4 and IFNA10 control DSS induced acute colitis through induction of Treg cells

**Figure 3:**
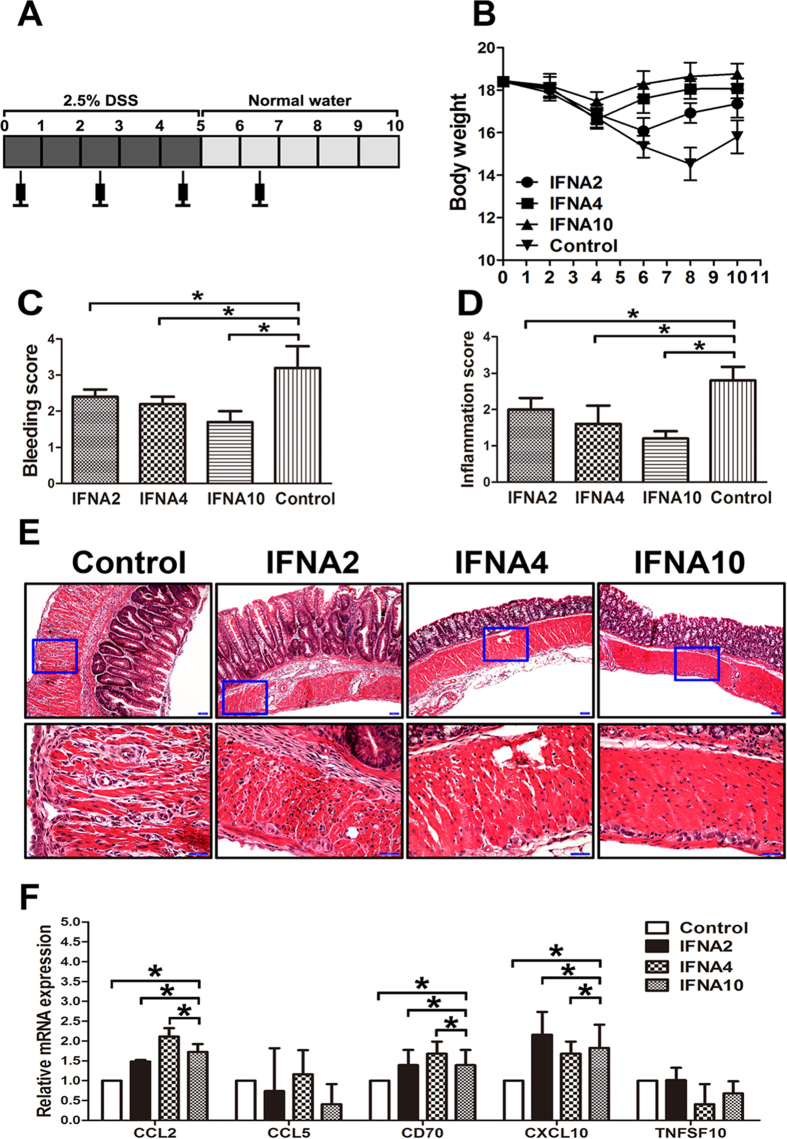
IFNA4 and IFNA10 control DSS-induced acute colitis. (**A-B**) Left panel showed the experimental protocol applied to induce mice acute colitis and the administration of IFNA subtypes; and right panel showed the mice body weight curve of DSS-induced acute colitis between the study groups. (**C**) The study mice were monitored for bleeding scores by a quantitative analysis for the presence of rectal blood. (**D-E**) Colon sections were fixed in formalin and stained with HE; the sections were analyzed for the inflammation score. (**F**) Real-time PCR analyzed the mRNA expressions of CCL2, CCL5, CD70, CXCL10 and TNFSF10 in colon tissues on DSS model. The data are given as the mean ± SD (n = 10, *P < 0.05).

To further evaluate the function of IFNA4 and IFNA10 during inflammation, we administrated recombinant IFNA4 and IFNA10 in a DSS-induced acute colitis model (Fig. 3A). As expected, the marked decrease in body weight, mucosal bleeding induced by acute inflammation, and histologic inflammation scores were significantly restored in the IFNA4 and IFNA10 treatment groups compared to the DSS-induced colitis controls; IFNA2 administration was applied as an IFN subtype control (Fig. 3A–D). In addition, the mRNA expressions of CCL2, CD70 and CXCL10 in colon tissues were upregulated after IFN administration on DSS model (Fig. 3F).

**Figure 4:**
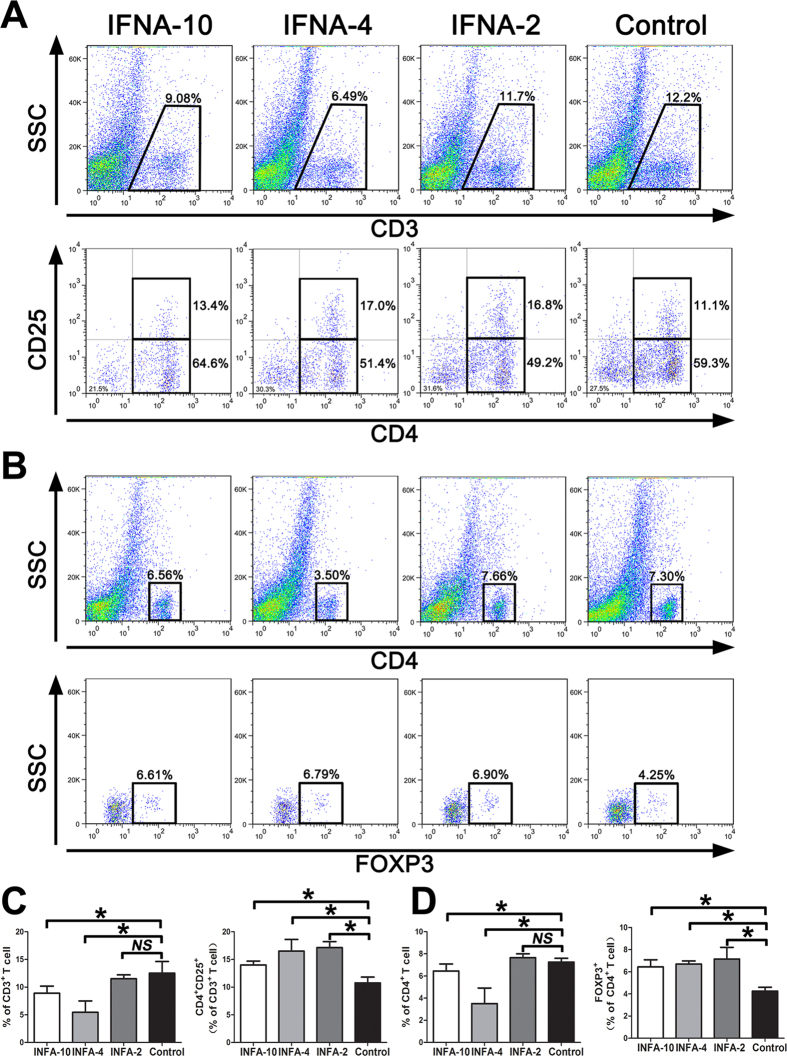
IFNA4 and IFNA10 inhibit acute colitis through induction of Treg cells. (**A-B**) FACS analysis of isolated cells from mice DSS-induce acute colitis groups, upper panels indicate first gate of lower panels. **(C-D)** Quantification analysis of upper FACS results, as CD4^+^ CD25^+^ cells in CD3^+^ and Foxp3^+^ cells in CD4^+^ T cells in the colon tissues, 5 mice in each group and analyzed the samples in triplicate, bars represent the mean ± SD.

Next, we analyzed the mechanism involved in restitution of acute colitis after DSS administration in the IFNA subtype treatment groups. As shown in Fig. 4, the total CD3^+^ inflammatory cells in the lamina propria were decreased, and CD4^+^ CD25^+^ and CD4^+^ Foxp3^+^ Treg cells were significantly increased in the IFNA subtype treatment groups compared to the control mice, indicating that different IFNA subtypes control acute colitis through the induction of Treg cells.

## Discussion

CD is a relapsing inflammatory bowel disease, mainly affecting the gastrointestinal tract, frequently presents with abdominal pain, fever, and clinical signs of bowel obstruction or diarrhea with the passage of blood, mucus, or both, and is associated with immune disorders. GWASs have identified susceptibility loci that are triggered by environmental factors, resulting in disturbed innate and adaptive immune responses toward a reduced diversity of commensal microbiota^26^. Epidemiological studies suggest that genetic factors play a significant role in CD patients and the clinical characteristics^27^. However, the variations associated with CD identified in Western populations may not be associated with CD susceptibility in Chinese patients of the Han population, suggesting the possible genetic heterogeneity of CD in different populations. Furthermore, the incidence of CD in China is increasing, placing a significant medical and economic burden on patients in recent years^28^.

In this study, we attempted to address the genetic heterogeneity of Chinese CD patients using exome sequencing, which allows the screening of the complete spectrum of variation within the protein coding sequences of the human genome. We uncovered 294 shared complete identical variants of whole-exon sequences in four CD patients, 26 of which were validated using Sanger sequencing. Confirmation in an additional 208 CD patients and 198 healthy individuals from three of medical center indicated that the heterozygous mutation frequency of IFNA10 (c.60 T > A) and IFNA4 (c.60 A > T) was significantly increased in the CD patients compared to the healthy controls. Furthermore, these variants are significantly associated with a resistance to steroid treatment in CD patients.

We further analyzed the characteristics of the IFNA10 variants and found that single-nucleotide changes (c.60 T > A) in exon 1 resulted in a cysteine to stop codon change before the signal peptide cleavage site, a mutation that might cause the loss of IFN subtype function. IFNs comprise a multigene family consisting of at least 13 IFNA subtypes, and the coding sequences of these genes diverge up to 8%, giving rise to 12 different functional subtype proteins. IFNA4 and IFNA10 are essential subunits of IFNA, and all IFNA subtypes are structurally similar and share a common cell surface receptor composed of IFNAR1 and IFNAR2^29^. By using CIPHER, a state-of-the-art network-based algorithm for genome-wide disease gene prediction^30^, we identified that IFNAR2, a receptor of IFNA4 and IFNA10, is strongly correlated with Crohn’s disease with a top rank at 65 (0.73%) among all 8919 genes (Supplementary Table 10). IFNs have various functions, such as anti-viral, anti-proliferative, anti-tumor, and immunomodulatory activities, and some directly inhibit viral transcription and translation and thus immediately reduce the loads of such viruses as chronic hepatitis B and C virus^31^,^32^. Our *in vitro* analysis showed that the IFNA4 and IFNA10 variants impaired the suppression of HCV replication in HuH7 cells.

The serum levels of IFNA10 were significantly decreased in the CD patients compared to the healthy controls and were also similarly decreased in the individuals with the variants compared to the wild-type allele in the CD and healthy control groups. Furthermore, the IFNA10 level was significantly decreased in the variant CD group compared to the variant control group. In contrast, the IFNA4 serum level was increased in the CD patients compared to the healthy controls and similarly increased in the individuals with the variants compared to the wild-type allele. We hypothesized the A was wild-type allele for Chinese people and T was the mutant in IFNA4 by opposite way. Thus, this phenotype is most likely induced from the single-nucleotide changes (c.60 A > T) resulted in the expression of the elongated IFNA4 protein and both of them was detected by ELISA kit.

The causes of IBD are most likely linked to both of genetic and microbiota-associated factors. Some reports reveal that CD and UC patients appear to harbor separate microbial communities, with a lower bacterial diversity compared to healthy individuals, and several bacterial groups have been suggested to be either increased or decreased in association with CD^33^,^34^,^35^,^36^. Lee *et al* showed that type I IFN signaling is important for maintaining Foxp3 expression in the inflamed mucosa in a model of T cell–mediated colitis and that the administration of recombinant IFNA ameliorates the severity of colitis by increasing the number of Foxp3 cells and by improving their function^37^. Our data from the mice model demonstrated that the marked decrease in body weight, mucosal bleeding induced by acute inflammation, and histologic inflammation scores were significantly recovered in the IFNA4 and IFNA10 treatment groups compared to the DSS-induce colitis controls; CD4^+^ CD25^+^ and CD4^+^ Foxp3^+^ Treg cells were also significantly increased in the IFNA subtype treatment groups compared to the control mice. Therefore, the function of IFNA4 and IFNA10 is potentially correlated with the intestinal microorganism environment of CD patients and should be further explored.

It is known that the binding of IFNA to the receptor complex initiates a signaling cascade through the activation of tyrosine kinase 2 and Jak 1 and the subsequent phosphorylation and dimerization of STAT1 and STAT2^32^. However, there is substantial biochemical evidence that individual IFN subtypes bind to different sites of IFNAR and have different binding affinities, leading to the formation of distinct signaling complexes^38^,^39^. We also explored the link between IFNA4/IFNA10 and known IBD susceptibility genes. According to the KEGG Pathway and Characteristic Subpathway Network Database (http://www.genome.jp/kegg/pathway.html and http://rulai.cshl.edu/tools/cipher/), IFNA4/IFNA10 and IFNR2^40^ are in the same pathway as the IL23R^5^ and IL10RA^41^ genes, which are associated with CD. However, as little is known to date about the biopathological mechanism of certain IFNA subtypes as genetic causes of CD, our findings provide a basis for future studies.

Although the application of exome sequencing to complex diseases is fraught with analytical difficulty^42^, our study revealed disease-associated gene variants in CD patients. We identified heterozygous IFNA10 and IFNA4 variants as a cause of impaired function and CD-susceptibility genes in Chinese individuals from multiple center based study. These findings provide clues for the understanding of the genetic heterogeneity of CD and may lead to better screening and improved treatment. Our results also highlight the value of applying exome sequencing to complex diseases, such as CD.

## Materials and Methods

### Ethics statement

This study was approved by the Ethics Committee (No: 20081009) of Zhongshan Hospital, affiliated to Xiamen University, Fujian Province, China. Written consent was obtained from all participants who were involved in the study. All procedures involving experimental animals were performed in accordance with protocols that were approved by the Committee for Animal Research of Xiamen University and complied with the Guideline for the Care and Use of Laboratory Animals (NIH publication No. 86-23, revised 1985).

### Patients

A total of 208 CD patients and 198 age and gender-matched healthy individuals undergoing a health examination at Zhongshan Hospital, Tenth People’s Hospital in Shanghai and Xijing Hospital in Xi’an from 15 September 2009 to 15 August 2014 were enrolled in this study. Supplementary Table 1 shows the clinical features of the four exome-sequenced CD patients. We obtained DNA and serum samples from the 208 CD patients and 198 healthy controls. Clinical and pathological information were obtained from patient’s database in Zhongshan Hospital affiliate to Xiamen University, Tenth People’s Hospital in Shanghai and Xijing Hospital of Digestive Diseases in Xi’an. The diagnosis of CD was based on standard clinical, endoscopic, radiological, and histological criteria^43^. Characteristics and genotypic details of 151 CD patients are summarized in Supplementary Table 8 and Table 9. The rest of CD patients are missed the standard follow-up and we do not included in the evaluation of therapeutic outcome. The clinical characteristics provided in the table are given according to the Montreal classification^44^.

### Exome capture

Genomic DNA was isolated from venous blood using TIANamp genomic DNA Kit (TIANGEN BIOTECH, Beijing, China) according to the recommended protocol. A 15-μg sample of genomic DNA from each of four patients affected with CD was sheared by sonication and individually solution hybridized with RNA probes (Agilent SureSelect human 38 M) to enrich the exonic DNA in each library. The array covered 18,837 (93.76%) of the total 20,091 genes in the Consensus Coding Sequence Region database 2009.

### Next-generation sequencing and data analysis

The exon-enriched DNA was sequenced using the Illumina Hiseq 2000 platform following the manufacturer’s instructions (Illumina, San Diego, CA). The raw image files were processed by the Illumina pipeline (version 1.3.4) for base calling and generating the read set, and the sequencing reads were aligned to the NCBI human reference genome (NCBI36.3) using SOAPaligner (soap2.21). Based on the results from SOAPaligner, SOAPsnp software was used to assemble the consensus sequence and call genotypes in the target regions. We collected reads that were aligned to the designed target regions for SNP identification and subsequent analysis. The consensus sequence and quality of each allele was calculated by SOAPsnp. Low-quality variations were filtered out using the following criteria: SNP quality equal to or larger than 20, sequencing depth larger than 4, estimated copy number no more than 2, and a distance between two SNPs larger than 5.

We aligned the reads to the reference genome by BWA and analyzed the alignment results with GATK to identify the breakpoints. Lastly, we annotated the genotypes of Insertions and deletions (indels).

Annotations of the variants were based on the NCBI and UCSC databases using ANNOVAR software with sorting intolerant from tolerant (SIFT) scores^45^. SIFT is a sequence homology-based tool that predicts whether an amino acid substitution is likely to affect the protein function; variants with SIFT scores of > = 0.05 are considered ‘benign’. All the nonsynonymous variants that were not predicted as benign by SIFT were classified as damaging, as were all the mutations in splice acceptor and donor sites and all coding indels.

### Sanger sequencing validation

The variants identified by exome sequencing were validated using ABI3730XL automated DNA Sanger sequencing. The primer sets are listed in Supplementary Table 5.

### Assessments of therapeutic efficacy

The efficacy of 5-aminosalicylate, corticosteroid, and infliximab (IFX) treatments were assessed at 3 months, and the immunosuppressant effect of AZA treatment was assessed at 6 months according to the modified classification^46^. The efficacy of medical treatment was defined as the resolution of CD-related symptoms with the discontinuation of medication or the improvement of symptoms with a reduction in the dose; inefficacy was defined as active symptoms without any reduction in medication dose, requiring an increase in dose and/or needing surgery^47^.

### Public data used

Data from dbSNP129 and 1000 Genome Project (20100208 release) were downloaded from the NCBI FTP site ( http://www.ncbi.nlm.nih.gov/Ftp/). Variants were characterized as novel if they were previously unreported in the dbSNP (v129) ( http://ftp.ncbi.nih.gov/snp/organisms/human_9606), 1000 Genomes Project ( www.1000genomes.org/, with the minor allele frequency, MAF > 1%^48^), HapMap 8 ( http://hapmap.ncbi.nlm.nih.gov/) and YH databases .

### Cell culture, RNA interfere and measurement of mRNA expression

The HuH7 and NCM460 cell lines were cultured in DMEM and RPMI 1640 supplemented with 10% fetal bovine serum (Life Technologies, Grand Island, NY), 1% penicillin G/streptomycin and incubated at 37℃ in an atmosphere of 95% air with 5% CO2 respectively. A single siRNA specifically target to highly homogeneous IFNA4 and IFNA10 genes was designed by RiboBio Co., Ltd. (RiboBio, Beijing, China). The target sequences for IFNA4 and IFNA10 siRNAs were: siRNA-1 (5′- CCTGGAAGCATGTGTGATA-3′), siRNA-2 (5′- GAGGTTGTCAGAGCAGAAA-3′) and siRNA-3 (5′- CCTGAAGGACAGACATGAT-3′), and the most efficient one (siRNA-1) was employed in this study. Total RNA was isolated from cells or colon tissue using TRIzol (Invitrogen, Carlsbad, CA) according to the manufacturer’s recommended protocol after 48 hours of transfection. The genes of IFNA4, IFNA10, FOXP3, TGFβ, IL10, IL2, IL6, IL17, CCL2, CCL5, CD70, CXCL10 and TNFSF10 mRNA expressions were measured using Real-time PCR (primers list as Supplementary Table 11).

### ***In Vitro*** Transcription, RNA infection, and Real time PCR

A full-length HCV JFH-1 strain plasmid was digested with a restriction enzyme, and the purified DNA template was used for RNA synthesis by *in vitro* transcription, essentially as previously described^49^. Briefly, the JFH-1 plasmid was transfected into 293FT cells and incubated at 37 °C for 12 hours; the medium was changed, and the cells were incubated for an additional 36 hours. The supernatant was harvested and immediately used to infect HuH-7 cells. We cloned the full-length IFNA4 and IFNA10 sequences based on the PLV plasmid and constructed mutant expression plasmids using the Quick-Change site-directed mutagenesis kit (Stratagene, La Jolla, CA). IFNA4, IFNA10, and the mutant plasmids were transfected into HuH-7 cells purchased from ATCC (American Type Culture Collection, Manassas, VA) using the QIAGEN transfection reagent (Qiagen, Valencia, CA). The HCV RNA copy number was quantified using real-time PCR. Total RNA (0.5 μg in a 50-μl reaction volume) was used with the quantitative TaqMan Real-time RT-PCR kit and an ABI Prism 7500 sequence detection system (Applied Biosystems, Foster City, CA). The following probe and primers were used for the quantification of the full-length JFH RNA: forward primer, 5′-CTGTCTTCACGCAGAAAGCG-3′; reverse primer, 5′-CACTCGCAAGCACCCTATCA-3′; and TaqMan probe, 5′-FAM-CATGGCGTTAGTATGAG-TGTCGTGCA-TAMRA-3′.

### Measurement of serum IFNA4 and IFNA10 level using ELISA

ELISAs were performed using the human IFNA4 and IFNA10 kit (for IFNA4, Catalog No. SEA175Hu; for IFNA10, Catalog No. SEG971Hu; USCN Life Science, Houston, TX, USA) according to the manufacturer’s instructions. Briefly, serum samples were diluted 1:4 in PBS, and the dilutions were added to a microplate and incubated for 2 h at 37 °C. Detection reagents A and B were added, followed by the substrate solution; the reaction was terminated with STOP solution. The absorbance was measured using a microplate reader at a wavelength of 450 nm.

### Mice colitis model

C57BL/6 mice were purchased from the laboratory animal center, Shanghai, China. The mice were maintained in a specific pathogen-free (including helicobacter-free and parvovirus-free) environment and were generally used between 6 and 8 weeks of age. A total of 2 μg of recombinant IFNA2, IFNA4, or IFNA10 protein (for IFNA2, Catalog No. 50525-M01H; for IFNA4, Catalog No. 50672-M08H; for IFNA10, Catalog No. 10349-H01H; Sino Biological, Beijing, China) diluted in 200 μl PBS was injected into the tail veins of C57BL/6 mice at days 1, 3, 5, and 7 of the acute colitis model. The study mice were treated with 2.5% (wt/vol) DSS (MP Biomedicals, Irvine, CA) for 5 days and then switched to normal drinking water. At day 10, the colon was harvested, with two pieces of tissue (0.5 cm in length) dissected, 2 cm away from the anus of each study mouse. One piece of colon tissue was fixed in 4% neutral buffered formalin, cut into 6-μm-thick sections, and then stained with hematoxylin and eosin (HE) for a light microscopic examination, and the other piece was prepared for cell isolation. All experiments were repeated at least three times.

### Histological Scoring of Inflammation

Colon tissues were removed from the mice, fixed with 4% paraformaldehyde, and embedded in paraffin. Sections from these samples were stained with hematoxylin and eosin (HE), and the degree of inflammation was graded semi-quantitatively on a scale from 0 to 6 in a blinded fashion, as previously described^50^. The inflammation score was composed of inflammatory cell infiltration ranging from 0 to 3 and tissue damage ranging from 0 to 3.

### Cell isolation and Flow cytometry (FCM) analysis

Colon tissue was collected from acute colitis mice and cut into pieces in 1 mg/ml of collagenase IV; collagenase digestion was performed in collagenase solution for 45 minutes at 37 °C. The eluted cells were then passed through a 70-μl cell strainer and collected for 10 minutes at 1400 rpm at room. The cells were incubated in 10% donkey serum and Fc blocked (BD Pharmingen, San Jose, CA) for 20 minutes at 4 °C and then stained with fluorescent-conjugated antibodies. FITC-conjugated CD3, APC-conjugated CD25, PE-conjugated CD4 (OX-38), and APC-conjugated Foxp3 were purchased from BD Pharmingen (San Diego, CA) and used for the FACS analysis. The cells were analyzed with the FACSCaliber cytometer (BD Bioscience, San Diego, CA) and analyzed using FlowJo software (Tree Star, Ashland, OR).

### Statistic Analysis

A statistic analysis was performed using SPSS 17.0 software (SPSS Inc., Chicago, IL). Comparisons of the data between the two groups were analyzed using the Wilcoxon two-sample test. The odds ratios (ORs) and 95% confidence intervals (CIs) were computed according to Woolf’s formula. The contribution of polymorphisms to the clinical features of the CD patients was also estimated by a chi-square (χ2) or Fisher’s exact test. All the values are expressed as the mean standard deviation (SD), and *P* < 0.05 was considered statistically significant. Each polymorphism was tested to ensure that it fit the Hardy-Weinberg equilibrium. The graphs were generated using Graphpad Prism 5.0 (Graphpad Software Inc., La Jolla, CA).

## Author Contributions

G.B., R.J.L., L.Z.J., N.Y.Z. and W.K.C. designed the experiments; X.C.X., X.H.Z., W.H.H., C.X., L.Y.S., L.P. and S.Y. collected the samples, performed the experiments and clinical data analysis; X.J.J. and L.S. contributed for informatics analysis, G.B. and X.C.X. wrote the paper and prepared the figures. All authors reviewed the manuscript.

## Additional Information

**How to cite this article**: Xiao, C.-X. *et al*. Exome sequencing identifies novel compound heterozygous IFNA4 and IFNA10 mutations as a cause of impaired function in Crohn's disease patients. *Sci. Rep.* **5**, 10514; doi: 10.1038/srep10514 (2015).

## Supplementary Material

Supporting Information

## Figures and Tables

**Table 1:** Genotype association study of the IFNA10 and IFNA4 variants in expanded CD cases versus controls.

**Locus**	**Groups**	**n**	**Genotype frequency [n(%)]**			***X***^***2***^	***P***
			**T/T (%)**	**T/A (%)**	**A/A (%)**		
IFNA4 (c.60 A > T)	CD	208	0 (0)	166 (79.81)	42 (20.19)	7.738	**0.005**
	Health group	198	0 (0)	134 (67.68)	64 (32.32)		
IFNA10 (c.60 T > A)	CD	208	48 (23.1)	160 (76.9)	0 (0)	11.902	**0.001**
	Health group	198	77 (38.9)	121 (61.1)	0 (0)		

**Table 2:** Association of IFNA4 variant with responses to medical treatment in CD Patients.

**Therapies**	**CD [n]**	**Efficacy**	**Genotype frequency [n (%)]**			***P***
			**T/T (%)**	**T/A (%)**	**A/A(%)**	
**5-ASA**	42	Effective 22	0	10 (45.4)	12 (54.6)	0.537
		Ineffective 20	0	11 (55.0)	9 (45.0)	
**Steroids**	28	Effective 8	0	2 (25.0)	6 (75.0)	**0.021**
		Ineffective 20	0	16 (80)	4 (20)	
**Immunosuppressant**	7	Effective 3	0	2 (66.0)	1 (34.0)	1.000
		Ineffective 4	0	2 (50.0)	2 (50.0)	
**IFX**	21	Effective 12	0	5 (41.6)	7 (58.4)	0.850
		Ineffective 9	0	5 (55.5)	4 (44.5)	
**Overall effects**	53	Effective 27	0	14 (51.9)	13 (48.1)	**0.004**
		Ineffective 26	0	23 (88.4)	3 (11.6)	

**Table 3:** Association of IFNA10 variant with responses to medical treatment in CD Patients.

**Therapies**	**CD [n]**	**Efficacy**	**Genotype frequency [n(%)]**			***P***
			**T/T (%)**	**T/A (%)**	**A/A(%)**	
**5-ASA**	44	Effective 20	8 (40.0)	12 (60.0)	0	0.911
		Ineffective 24	10 (41.7)	14 (58.3)	0	
**Steroids**	27	Effective 10	6 (60.0)	4 (40.0)	0	**0.027**
		Ineffective 17	2 (11.8)	15 (88.2)	0	
**Immunosuppressant**	9	Effective 5	2 (40.0)	3 (60.0)	0	1.000
		Ineffective 4	2 (50.0)	2 (50.0)	0	
**IFX**	24	Effective 14	6 (42.9)	8 (57.1)	0	0.889
		Ineffective 10	4 (40.0)	6 (60.0)	0	
**Overall effects**	47	Effective 24	13 (54.2)	11 (45.8)	0	**0.008**
		Ineffective 23	3 (13.1)	20 (86.9)	0	
