# Supplementary material for: Exome sequencing identifies novel compound heterozygous IFNA4 and IFNA10 mutations as a cause of impaired function in Crohn’s disease patients
Source: Sci Rep. 2015 May 22;5:10514. doi: 10.1038/srep10514 (PMC4441321; doi:10.1038/srep10514)
Supplement: Supporting Information [file srep10514-s1.pdf]

Exome sequencing identifies novel compound heterozygous IFNA4 and IFNA10 mutations as a cause of impaired function in Crohn's disease patients

Chuan-Xing Xiao, Jing-Jing Xiao, Hong-Zhi Xu, Huan-Huan Wang, Xu Chen, Yuan-Sheng Liu, Ping Li, Ying Shi, Yong-Zhan Nie, Shao Li, Kai-Chun Wu, Zhan-Ju Liu, Jian-Lin Ren and Bayasi Guleng

Supplementary Table 1 Summary of the clinical features of four CD patients by exome sequencing

Sample	Case 1	Case 2	Case 3	Case 4
Gender	F	M	M	M
Age at exam (years)	58	36	20	29
Age at onset (years)	56	34	18	28
Disease localization	Ileal	Ileal	Ileal	Ileal ,colonic
Disease Behavior	Stricturing, Perianal disease	Stricturing	Stricturing	Stricturing
Extraintestinal manifestations	Canker sore， Arthritis	-	Biliary calculus	-

Supplementary Table 2 Overview of the data production for the exome sequencing of four individuals with CD

Exome Capture Statistics	Case 1	Case 2	Case 3	Case 4
Target region (bp) (1)	37,563,132	37,640,396	37,640,396	37,640,396
Raw reads	68,220,612	60,154,458	60,255,526	64,838,622
Raw data yield (Mb)	6,140	5,414	5,423	5,835
Reads mapped to genome	60,497,797	54,240,309	54,688,801	58,816,288
Reads mapped to target region (2)	39,309,688	35,563,945	37,816,485	39,119,514
Data mapped to target region (Mb)	2,938.65	2,657.93	2,842.73	2,937.04
Mean depth of target region (X)	78.23	70.61	75.52	78.03
Coverage of target region (%)	98.01%	98.02%	97.82%	97.77%
Average read length (bp)	89.87	89.89	89.91	89.90
Rate of nucleotide mismatch (%)	0.34	0.35	0.34	0.34
Fraction of target covered >=4X (%)	94.18	94.16	93.69	93.57
Fraction of target covered >=10X (%)	87.26	87.07	86.44	86.21
Fraction of target covered >=20X (%)	77.64	76.90	76.55	76.33
Fraction of target covered >=30X (%)	69.67	68.22	68.32	68.25
Fraction of target covered >=40X (%)	62.57	60.4	61.06	61.21
Capture specificity (%) (3)	67.63	68.54	72.14	69.46
Reads mapped to flanking region (4)	5,782,431	5,850,622	4,800,361	5,180,738
Mean depth of flanking region	19.03	18.00	16.92	17.86
Coverage of flanking region (%)	91.94	92.43	90.22	90.80
Fraction of flanking covered >=4X	73.27	74.79	68.94	70.41
Fraction of flanking covered >=10X	49.81	50.71	45.00	46.60
Fraction of flanking covered >=20X	30.51	29.73	26.85	28.13
Fraction of flanking covered >=30X	20.26	18.87	17.56	18.6
Fraction of flanking covered >=40X	13.92	12.43	11.93	12.79
Fraction of unique mapped bases on or near target (%)	77.10	79.09	80.90	78.23
Duplication rate (%) (5)	7.68	6.40	5.82	5.67
Mean depth of chrX	97.78	45.31	48.60	49.34
GC rate (%)	43.06	43.36	43.14	42.88
Gender test result	F	M	M	M

(1) Target regions here refer to the regions that are actually covered by the designed probes.

(2) Reads mapped to target regions are reads that within or overlap with target region.

(3) Capture specificity is defined as the percentage of uniquely mapped reads aligning to target region.

(4) Flanking region refers to regions +/-200 bp on both sides of each target region.

(5) Duplication is defined as pairs of reads that have duplicated start sites for both reads. Duplication rate is the fraction of duplicated reads in raw data.

Supplementary Table 3 Summary of SNPs for exome capture

Categories	Case 1	Case 2	Case 3	Case 4
Number of genomic positions for calling SNPs[(1)]	105,924,243	106,141,607	106,141,607	106,141,607
Number of high-confidence genotypes [(2)]	93,388,782	94,432,231	91,733,793	92,372,962
Number of high-confidence genotypes in target regions	36,392,476	36,443,972	36,348,257	36,337,035
Total number of SNPs	62,091	63,581	58,392	59,449
Synonymous-coding	8,008	8,077	7,921	7,964
Missense	6,879	7,013	6,723	6,741
Nonsense	61	57	56	53
Readthrough	6	6	6	6
Splice site [(3)]	387	406	369	372
Intron	43,310	44,513	40,129	40,992
5' UTRs	744	743	666	711
3' UTRs	2,010	2,110	1,900	2,013
Intergenic	686	656	622	597
hom	25,223	26,623	24,645	25,047
het	36,868	36,958	33,747	34,402
FrameError	0	0	0	0

(1) Genomic positions for calling SNPs are defined as all the positions in *.cns file, which include capture target regions and its 200bp flanking regions.

(2) Consensus genotype with quality score of at least 20.

(3) Intronic SNPs within 4bp of exon/intron boundary.

Supplementary Table 4 Filtration of SNP/InDels from Exome sequencing data

Feature_SNP/InDels	Case 1	Case 2	Case 3	Case 4
Functional_SNPs/InDels	9604	9691	9332	9342
Filtered_DBsnp	2637	2632	2637	2659
Filtered_DBsnp_1000gene	1695	1681	1652	1672
Filtered_DBsnp_1000gene_ Hapmap	1693	1680	1650	1671
Filtered_DBsnp_1000gene_ Hapmap_YH	1648	1632	1602	1623
Share_all_cases			294	
Filtered_Prediction from SIFT			99	
Filtered_ Function of Gene			57	
Sanger sequence for validation			26	

Supplementary Table 5 Primers of 57 candidate genes used for Sanger sequencing validation

	Gene	Primers 5'-3'	
		Forward	Reverse
1	NOD2	CCTTTGATGGCTTTGACG	GCTGGGCTGAGAACACG
2	DLG5	ATGGAGCCGTTCAAGTAAA	AAGGAGCACAAAGCAGACA
3	HLA-A	CATTGGGTGTCGGGTTTCC	CGGCCTCGCTCTGGTTGTAG
4	HLA-A	GGGTGTCCTGTCCATTCTCA	TCTTCCTCCTCCACATCACG
5	HLA-A	CCATCGTGGGCATCATTG	CATCAGCCTCCTTCATACTTACTT
6	HLA-A	AAGTCGCTGTTCCCTTCTCA	CCCATCTCCCTCCTTACCC
7	MICB	TGGAGCATTTACCCGTTTC	TTCCACAGCCCTTCGTAT
8	MTHFD1L	ATTGCTGCCGAGACTGT	GAGGGTGAGGACTGCTTT
9	TNFRSF19	ATGGTGCCGACTTTCTT	GCTGTCCACTTTGCTCTT
10	ATRNL1	GGGACATTCAGCACATA	CCAAAGAAATAGCACCA
11	ATRNL1	GAGGGACATTCAGCACA	CCAAAGAAATAGCACCA
12	IFNA10	CCCAAGCAGCAGATGAG	TTGAGTGCAGGTGAAAA
13	PMS2P3	TCTCCCTGCTCCACTTC	TTTCCCTTCCATAATCTGT
14	TUBA3E	TTCCGTCAAGTCCACATTCA	GCACTCTAACCTGGGCAACA
15	IFNA4	CAAGCAGCAGATGAGTC	CTTTGAGTGCAGGTGAA
16	BCLAF1	GTGGGTGCAAGTTCTG	CAAATGATTGTCTGGGT
17	IPO5	GCTCTTTCGCCCAGAC	GGCACCCTTCACCAAT
18	RND3	AAGTCCACAGGGCAAGG	TCACTCAGTCATGGGCATC
19	SNORD114-2	AGACTGGTGTTAGGGATA	TCTTGCCTGACCACAT
20	ZNF518B	CATACATACCGTCCTTCC	GGCTTACTTGCCAACTCT
21	MST1	CCTAAGTGGGCAATGTCTATG	GGGAGGCAGTGTTGAAGC
22	SLC35E2	TCCACTTGCCTTGTCCT	TGGTGATGTTCAGCCTTT
23	MST1	GCTAGGCATTCAGACTCCA	GCCACCCAATCCCGTA
24	LRRC37A2	AGCATCGGTAGGGAATC	GGAAATAGCGTGGGTT
25	THOC3	ATGCCACAAAGTCTACCA	GCTCAGTGACCTGCTCTT
26	SON	TTTGCAGTCCCTTTCC	GCCATGAGGTCCTTTG
27	OR2B11	CGTGGTGGTGTAGCAGAG	TGACGGTGATGGGAGC
28	RNF145	TCCAGCAGGCGGTAA	TTGCGAGTTGTAGAACAG
29	MYH15	CCTCTTCTGTGGGTACTTC	CACTGTGCCTTCTATGTCTA
30	QRICH2	GTGGATCTGCCTGAGGTT	GTGATTTGGCTCAACCTG
31	GPATCH4	GAGCCCTACCTTTACCAG	GCAAACCAATCAGTCCC
32	AICDA	AAAAGCTGATGTGGAGGAG	TGCGGAATGAATGAGTTAG
33	GALC	AAATGCTTCTACCACCAA	GAAACGCCATTGACC
34	CCDC66	AACTGATAGGAATGTGGGTA	GAAATAGAAAGTGGCAAGTA
35	hCG_17324	CATTCTCCCAGGTTTGC	CGATTCTCCACCACGA
36	HRNR	GACAAGAAGGTGGAAAGG	GGTAATGGCAGGAAGC
37	MYLK	AAGACACCTGGCACTGGA	TGGGTGGCAAGAAGAAAT
38	SIGLEC12	CTCATCACATTTGCCTTTG	CTACTGCTGCTGCTACTGC
39	TCEAL6	TGCTGGTAACGAGATTGA	CTGGAAGATAAGGGAAGC
40	'OR2T3	CTCATCCTCCTCATCCACTCAG	ATGATGGGGGCGAGAAGC

41	'OR2T34	TTGCGGAGACTGTAAATGAGG	TCTCCATTACCCACTGCTGAT
42	'hCG_17324	TTGCCTCTGGGAACCATAAAT	GACCACTGAAGCCTTCCAAG
43	'HSPD1	GCTCTTGTACCCAGTATCAGG	GTAGAGACGGGGTCTCATCATG
44	'MYH15	CTTCTGTGGGTACTTCCATTCCTA	TGTGCCTTCTATGTCTAGTGGGTA
45	'C3orf17	GGATAATACGTGCTGAACAGTGA	TGGGAGTGACTGGTGTGACAT
46	'NHEDC1	TGCCAAATTAAGAAGCAGAGACT	TAATGGACATTTCTGAGTTGTGTG
47	'ANLN	ATGCAGACTAACCCACATATTATGA	ATTCTCCCTTGGATGGAACTG
48	'PCMTD1	CCACTATAATTTGCTCTGATGAAAG	CTGTGCTGTCAGGAATCTACAGG
49	'GPRIN2	ACAGTGGTGCTCGGAAGGC	TGGGACTAACCCAGCGGGAC
50	'PRKRIR	TATTTCCCGTTACATTACC	GTCAGTCTACTATTTCCAA
51	'ARHGAP5	TGAGCACTTTAGAAGCTG	CCCACTAAAGAAACATGTCTT
52	'WDR22	CTCACGTCTCACTAGAAAGG	TGTCTGAGTGTCCTGGGGAA
53	'CHRFAM7A	CAAAATTCAATAAATGCTCGC	CAGAATCTCAGCTAAGCTTCAC
54	'CDC27	CTACAGTATCTGCAAAAGTT	CAAGCCTCAACTTAGAACAGA
55	'CDC27	AAAGGGAATCAGAGTTTAA	GACAATCATGGTATTCTCTGT
56	'QRICH2	CACGCTGGTCCATTCCAGGT	AACCACGCTGATCTACTCCAG
57	'ZNF285A	GGCAGTGTCCCTGTCTT	TTTAGTTCAAGCTCCGTTC

Supplementary Table 6 Summary of the 26 shared variants in all four patients with CD

No	Chromosome	Position	Reference	Alteration	MutType	GeneName
1	chr6	30020087	G	C	SNP	HLA-A
2	chr6	31585750	G	A	SNP	MICB
3	chr9	21197037	T	A	SNP	IFNA10
4	chr7	74983431	G	A	SNP	PMS2P3
5	chr9	21177471	A	T	SNP	IFNA4
6	chr13	97452556	C	T	SNP	IPO5
7	chr3	49701032	T	C	SNP	MST1
8	chr3	49701032	T	C	SNP	MST1
9	chr17	41981244	C	A	SNP	LRRC37A2
10	chr5	175319745	C	T	SNP	THOC3
11	chr1	245681885	-1A	-1A	Indel	OR2B11
12	chr5	158521212	-5AAAAA	-5AAAAA	Indel	RNF145
13	chr3	109712127	N/A	+1A	Indel	MYH15
14	chr1	154831673	N/A	+2AC	Indel	GPATCH4
15	chr12	8648789	N/A	+2AC	Indel	AICDA
16	chr14	87486849	-1A	-1A	Indel	GALC
17	chr3	56625094	N/A	+3CTC	Indel	CCDC66
18	chr2	120121100	-1T	-1T	Indel	hCG_17324
19	chr1	150462353	-1T	-1T	Indel	HRNR
20	chr19	56696603	N/A	+1C	Indel	SIGLEC12
21	chr1	246803966	C	T	SNP	'OR2T34
22	chr2	120121100	-	'-T	Indel	'hCG_17324
23	chr3	114215000	-	'-T	Indel	'C3orf17
24	chr7	36413874	-	'+CTT	Indel	'ANLN
25	chr10	46419597	-	'+ATGAGG	Indel	'GPRIN2
26	chr14	68590272	-	'-T	Indel	'WDR22

Supplementary Table 7 Summary of two verified variants in CD cases

Chr	Position	Name	Ref	Alt	M T	PFS	M N	SFS	Codons	Sub
chr9	21177471	IFNA4	A	T	SNP	Damaging	c.60 A>T	0	TGA60TGT	C20*
chr9	21197037	IFNA10	T	A	SNP	Damaging	c.60 T>A	0	TGT60TGA	C20*

Chr: Chromosome; Ref: Reference; Alt: Alteration; MT: Mutation Type; PFS: Prediction from SIFT; MN: Mutation Nomenclature; SFS: Score from SIFT; Sub: Substitution

Supplementary Table 8 Genotype-phenotype association analysis for IFNA4 with disease characteristics in CD Patients

Characteristics	CD [n]	Genotype frequency [n(%)]			*P*
		T/T	T/A	A/A	
Gender					
Male	76	0	63	13	0.854
female	75	0	60	15	
Age at diagnosis (yr)					
20 yr or younger,	16	0	10	6	0.079
Over 20 yr,	135	0	112	23	
Disease localization in CD					0.176
Terminal ileum, L1	33	0	27	6	
Colon, L2	52	0	45	7	
Ileocolon, L3	44	0	30	14	
Upper GI, L4	22	0	18	4	
Disease Behavior					
Stricturing	23	0	18	5	1.000
Non-stricturing,	33	0	27	6	
Penetrating	14	0	10	4	0.680
Non-penetrating,	32	0	28	4	
Perianal disease,	18	0	12	6	0.603
No Perianal disease	31	0	26	5	

Supplementary Table 9 Genotype-phenotype association analysis for IFNA10 with disease characteristics in CD Patients

Characteristics	CD [n]	Genotype frequency [n(%)]			*P*
		T/T	T/A	A/A	
Gender					0.638
Male	84	15	69	0	
female	67	14	53	0	
Age at diagnosis (yr)					0.226
20 yr or younger,	16	2	14	0	
Over 20 yr,	135	27	108	0	
Disease localization in CD					0.305
Terminal ileum, L1	31	6	25	0	
Colon, L2	61	12	49	0	
Ileocolon, L3	38	8	30	0	
Upper GI, L4	20	3	17	0	
Disease Behavior					
Stricturing	17	4	13	0	1.000
Non-stricturing,	36	8	26	0	
Penetrating	12	3	9	0	1.000
Non-penetrating,	39	7	28	0	
Perianal disease,	16	2	14	0	1.000
No Perianal disease	31	5	26	0	

Supplementary Table 10. The top 100 genes of Crohn's disease predicted by CIPHER.

MIM:266600: INFLAMMATORY BOWEL DISEASE 1; IBD1

Gene	UniProtID	RefSeqID	Rank
IL10RA	Q13651	NP_001549	1
NOD2	Q9HC29	NP_071445	2
NLRC4	Q9NPP4	NP_067032	3
IL23R	-	NP_653302.2	4
IL23A	-	NP_057668.1	5
SLC22A4	-	NP_003050.2	6
IL10RB	Q08334	NP_000619	7
-	Q9BUU4	-	8
IL28A	-	NP_742150.1	9
IL28A	Q8IZJ0	NP_742150	10
IL28B	Q8IZI9	NP_742151	11
IL29	Q8IU54	NP_742152	12
IL10	P22301	NP_000563	13
UCN2	Q96RP3	NP_149976.1	14
IL28B	-	NP_742151.2	15
IL22	Q9GZX6	NP_065386	16
IL22RA1	-	NP_067081.2	17
IL22RA2	Q969J5	NP_443194	18
-	Q6N095	-	19
ERBB2IP	Q96RT1	-	20
SLC22A5	O76082	NP_003051.1	21
SLC26A6	Q9BXS9	NP_075062.1	22
PDZK1IP1	-	NP_005755.1	23
SLC22A12	-	NP_653186.2	24
SLC17A1	Q14916	NP_005065.1	25
PDZK1	-	NP_002605.2	26
RIPK2	O43353	NP_003812	27
-	Q969D9	-	28
-	-	NP_149024.1	29
CARD6	Q9BX69	NP_115976	30
IL1RAP	-	NP_002173.1	31
STAT3	P40763	NP_998827	32
IL1RN	P18510	NP_776215	33
IL1RAP	Q9NPH3	NP_608273	34
JAK2	O60674	NP_004963	35
IL12RB1	P42701	NP_005526	36
JAK1	P23458	NP_002218	37
IL18R1	Q13478	NP_003846	38
NDUFA13	Q9P0J0	NP_057049.3	39

STMN1	P16949	NP_005554.1	40
NLRP1	Q9C000	NP_127497	41
NALP4	Q96MN2	NP_604393.1	42
GATA2	P23769	NP_116027.2	43
IL21R	Q9HBE5	NP_851565	44
EHMT1	Q9H9B1	NP_079033	45
-	Q7RTZ4	-	46
UCN3	Q969E3	NP_444277.2	47
IL21	Q9HBE4	NP_068575	48
-	Q8IZ56	-	49
IL21	-	NP_068575.1	50
BHLHB2	O14503	NP_003661	51
KPNA1	P52294	NP_002255	52
FGFR4	P22455	NP_998812	53
PRKDC	P78527	NP_008835	54
SH2D2A	Q9NP31	NP_003966	55
STAT5A	P42229	NP_003143	56
SMARCE1	-	NP_003070.3	57
CCR1	P32246	NP_001286	58
IL1R1	P14778	NP_000868	59
ATM	Q13315	NP_612149	60
BRCA1	P38398	NP_009228	61
IL1F10	Q8WWZ1	NP_115945.4	62
CLCN3	P51790	NP_776297.2	63
SLC22A11	-	NP_060954.1	64
IFNAR2	P48551	NP_997468	65
TYRO3	Q06418	NP_006284	66
MAP3K11	-	NP_002410.1	67
LCK	P06239	NP_005347	68
DDX54	Q8TDD1	NP_076977	69
TRAT1	-	NP_057472.2	70
FADD	Q13158	NP_003815	71
FCGR2A	P12318	-	72
JUNB	P17275	NP_002220	73
IGHM	P01871	CAA34971.1	74
STAT1	P42224	NP_644671	75
KIFC1	Q9BW19	NP_002254	76
TREX1	Q9NSU2	NP_569055.1	77
TOP3A	Q13472	NP_004609	78
MSH6	P52701	NP_000170	79
RECQL	P46063	NP_116559	80
MSH2	P43246	NP_000242	81
IKBKB	O14920	NP_001547	82
ZNF467	-	NP_997219.1	83

HUS1	-	NP_004498.1	84
ADAM12	O43184	NP_003465	85
-	Q9ULE1	-	86
KIR2DL3	P43628	NP_056952.2	87
MAGEA12	P43365	NP_005358	88
DOCK1	Q14185	NP_001371	89
ELMO3	Q96BJ8	NP_078988	90
-	Q8N3K4	-	91
-	Q7Z5G9	-	92
BTK	Q06187	NP_000052	93
IL6R	P08887	NP_000556	94
PIK3R1	P27986	NP_852664	95
-	Q13901	-	96
ITK	Q08881	NP_005537	97
ANKRD11	-	NP_037407.4	98
CHAF1A	Q13111	NP_005474	99
DOK1	-	NP_001372.1	100

Supplementary Table 11 Primers of the genes used for Real-time PCR

	Gene	Primers 5'-3'	
		Forward	Reverse
1	IFNA4	GTTCCAGAAGGCTCAAGCCATC	TAGGAGGCTCTGTTCCCAAGCA
2	IFNA10	CCCAAGCAGCAGATGAG	TTGAGTGCAGGTGAAAA
3	FOXP3	GGCACAATGTCTCCTCCAGAGA	CAGATGAAGCCTTGGTCAGTGC
4	TGF-b	TACCTGAACCCGTGTTGCTCTC	GTTGCTGAGGTATCGCCAGGAA
5	IL-10	TCTCCGAGATGCCTTCAGCAGA	TCAGACAAGGCTTGGCAACCCA
6	IL-2	AGAACTCAAACCTCTGGAGGAAG	GCTGTCTCATCAGCATATTCACAC
7	IL-17	CGGACTGTGATGGTCAACCTGA	GCACTTTGCCTCCCAGATCACA
8	IL-6	AGACAGCCACTCACCTCTTCAG	TTCTGCCAGTGCCTCTTTGCTG
9	CCL2	AGAATCACCAGCAGCAAGTGTCC	TCCTGAACCCACTTCTGCTTGG
10	CCL5	CCTGCTGCTTTGCCTACATTGC	ACACACTTGGCGGTTCTTTCGG
11	CD70	TTCGCACAGGCTCAGCAGCAG	TTGTCCAGCTCTGGTCCATGCA
12	CXCL10	GGTGAGAAGAGATGTCTGAATCC	GTCCATCCTTGGAAGCACTGCA
13	TNFSF10	TGGCAACTCCGTCAGCTCGTTA	AGCTGCTACTCTCTGAGGACCT

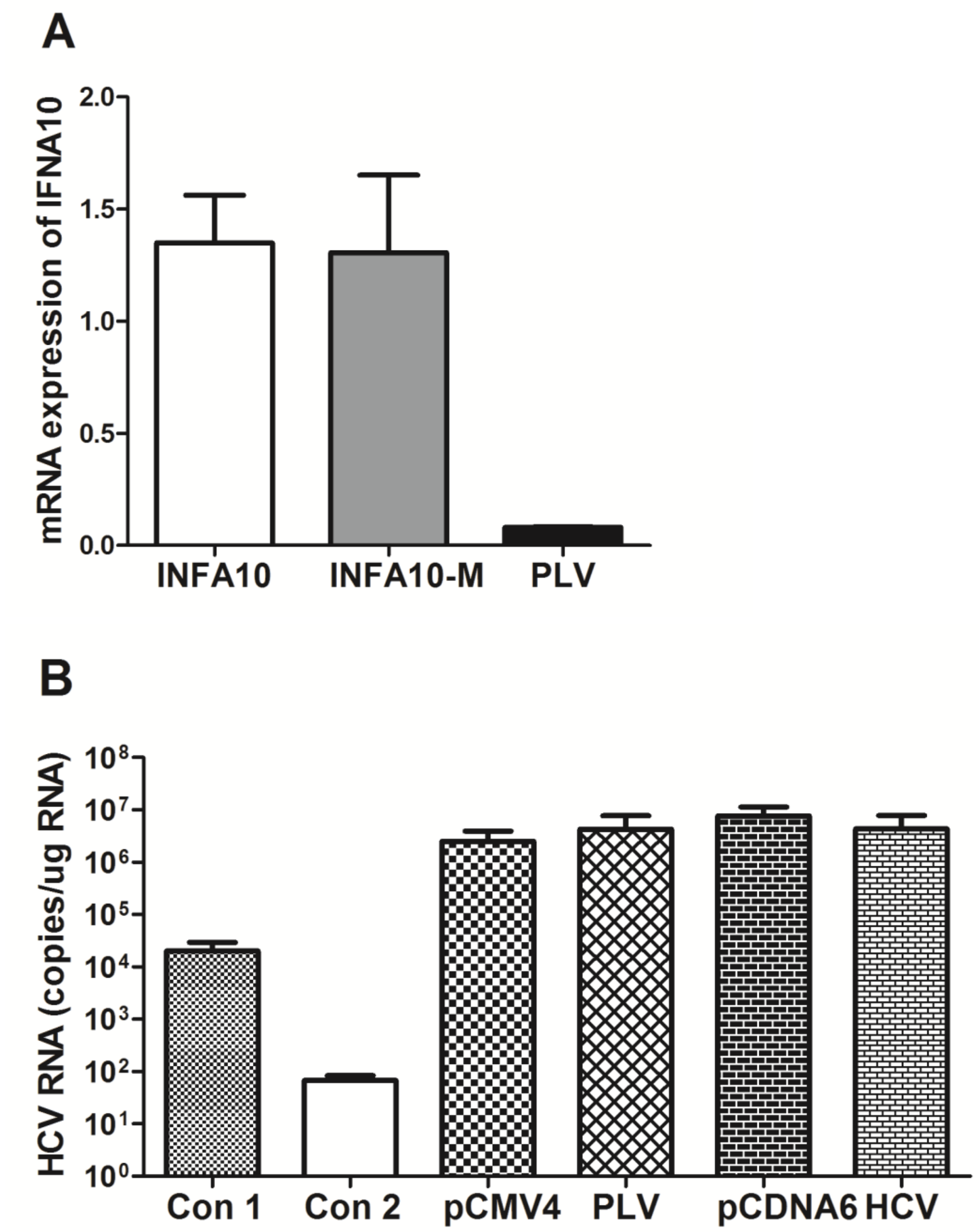

Figure legend

Supplementary Figure 1. RT-PCR analysis of relative mRNA expressions. (A) Huh7 cells were transiently transfected with plasmids expressing IFNA10 or mutants; mRNA expressions were analyzed using real-time PCR and PLV as a control. (B) The HCV plasmid (HCV) and empty plasmids (pCMV4, PLV and pCDNA6) were transiently co-transfected to HuH7 cells, without the transfection reagent (Con 1) and transfection reagent only (Con 2) were applied as controls; the replicon copies of HCV were calculated using real-time PCR.
